# Implementation of home measurement of CRP levels in diagnosis and monitoring of children with autoinflammatory diseases

**DOI:** 10.1186/1546-0096-13-S1-P159

**Published:** 2015-09-28

**Authors:** B Wolska-Kuśnierz, B Mikołuć, E Bernatowska

**Affiliations:** 1 CMHI, Immunology, Warsaw, Poland; 2 Medical University, Department of Pediatrics and Developmental Disorders of Children and Adolescents, Białystok, Poland

## Introduction

Recognition of systemic autoinflammatory diseases (SAIDs) in the last decade is growing rapidly. About 250 children is under care with suspicion or diagnosis of SAIDs in our centre and monitoring of inflammatory markers is fundamental in their diagnosis and monitoring of treatment.

## Objective

To check the practical usefulness of home CRP monitoring in patients during diagnosis or monitoring of autoinflammatory diseases.

## Material

Ten patients with genetically confirmed or suspicion of AIDs (2 FMF, 2 TRAPS, 1 MWS, 2 PFAPA, 3 suspicion of SAIDs) were monitored at home with both use of diary of symptoms/treatment and regular CRP measurement.

CRP levels were checked by parents at home using ready-to-use system on a finger-prick blood sample of their child.

## Results

Ready-to-use system provided patients with very fast, easy to perform and safe CRP measurement, having obtained reliable results.

Children and their parents found avoidance of frequent outpatient clinic visits and painful collection of blood from veins extremely important. Parents were able to use the system properly just after 2-3 hours of practical training.

Regular, up to twice a day measurement of CRP greatly facilitated the diagnosis and monitoring of patients' treatment. It proved to be extremely useful in an appropriate modification of treatment with the use of steroids in PFAPA or IL1-blockers in TRAPS.

## Conclusions

The results of introductory pilot study of home measurement of CRP levels in children with autoinflammatory diseases are encouraging. The opportunity of fast, regular monitoring of inflammatory marker in home conditions improved both diagnostic-therapeuthical process as well as quality of children's life.

